# Evaluation of the Effect of Alpha2-Adrenergic Receptor Stimulation on Prolactin Secretion Using the Clonidine Test in the Diagnosis of Children with Short Stature

**DOI:** 10.3390/ijms26209939

**Published:** 2025-10-13

**Authors:** Angelika Pakuła, Anna Fedorczak, Marzena Kolasa-Kicińska, Anna Łupińska, Maciej Hilczer, Arkadiusz Zygmunt, Renata Stawerska

**Affiliations:** 1 Department of Endocrinology and Metabolic Diseases, Polish Mother’s Memorial Hospital—Research Institute, 93-338 Lodz, Poland; angelika.pakula@iczmp.edu.pl (A.P.); renata.stawerska@iczmp.edu.pl (R.S.); 2 Department of Pediatric and Adult Endocrinology, Medical University of Lodz, 90-419 Lodz, Poland

**Keywords:** prolactin, alpha2-adrenergic receptors, clonidine, growth hormone, idiopathic short stature, growth hormone deficiency, children

## Abstract

Prolactin (PRL) and growth hormone (GH) originate from somatomammotropic cells and share regulatory mechanisms. Alpha_2_-adrenergic receptor stimulation with clonidine is routinely used in diagnosing GH deficiency (GHD), yet its effect on PRL secretion remains unclear. This study aimed to assess the impact of clonidine-induced α_2_-adrenergic receptor stimulation on PRL secretion and compare PRL dynamics between children with idiopathic short stature (ISS) and GHD. Forty-nine children with height < −2.0 SD (29 ISS, 20 GHD) underwent clonidine stimulation (0.15 mg/m^2^ administered orally). Serum GH and PRL were measured at 0, 30, 60, 90, and 120 min. Groups did not differ in chronological age, bone age, height SDS, or BMI SDS. Both groups exhibited a significant decrease in PRL at 30, 60, and 90 min compared to baseline. In ISS, PRL concentrations increased from 60 min onward, returning near baseline at 120 min. In GHD, PRL remained suppressed throughout the test. GH and PRL concentrations correlated positively at 90 (r = 0.35, *p* < 0.05) and 120 min (r = 0.35, *p* < 0.05). Clonidine-induced alpha2-adrenergic stimulation suppresses PRL in both ISS and GHD, but recovery is observed only in ISS, suggesting a potential involvement of GH signaling in PRL regulation.

## 1. Introduction

Prolactin (PRL)-producing lactotroph cells and growth hormone (GH)-producing somatotroph cells, located in the pituitary gland, are ontogenically derived from progenitor cells (somatomammotrophs). Both GH and PRL receptors are members of the Class I cytokine receptor superfamily. These receptors share structural homology and a common signaling mechanism involving activation of the tyrosine kinase JAK2 and downstream STAT pathways [1,2]. The overlap in receptor family membership and intracellular signaling cascades provides a molecular basis for functional cross-talk between the GH and PRL axes. Their pleiotropic effects contribute to (but are not limited to) the regulation of the most essential vital processes of the human species: growth (GH) and reproduction (PRL) [2]. PRL secretion is regulated primarily by the inhibitory action of dopamine via D2 receptors and the stimulatory action of thyrotropin-releasing hormone (TRH) and estrogens [3]. In addition, PRL secretion shows a circadian rhythm with nocturnal increases associated with sleep onset [3,4]. Other modulators such as serotonin, oxytocin, and vasoactive intestinal peptide have also been described [5]. Recent studies suggest that there are functional interactions between GH and PRL secretion, reflecting their common ontogeny and overlapping regulatory pathways. [6,7]. The role of other factors, particularly the adrenergic system, is not fully understood. 

Stimulation of the alpha2-adrenergic receptor is widely used in the diagnosis of growth hormone deficiency (GHD) in children. Clonidine, the alpha2-adrenergic agonist, has been used as a GH secretion-stimulating agent since the 1970s and is still used as a pharmacological screening tool in GHD [8,9]. Simultaneously with stimulation of presynaptic alpha2-adrenergic receptors, clonidine enhances the secretion of somatoliberin (growth hormone-releasing hormone, GHRH) from the hypothalamus, thereby promoting GH secretion [10]. According to Obara et al., the accuracy of the clonidine stimulation test in GHD is 72%, with a sensitivity of 50% and a specificity of 86.7% [11]. On the other hand, it appears that presynaptic stimulation of the alpha2-adrenergic receptor inhibits the secretion of noradrenaline (norepinephrine). It is hypothesized that this suppression may simultaneously inhibit PRL secretion, although the underlying mechanisms remain unclear. Given the long-standing concerns regarding the low reproducibility of GH stimulation tests and the ongoing debate over the GH cut-off threshold distinguishing GHD from normal GH secretion in children, we aimed to investigate whether PRL levels change during the stimulation test and whether such changes influence GH secretion. Measuring PRL during clonidine stimulation may provide complementary information on hypothalamic–pituitary regulation. A blunted or absent PRL recovery could indicate broader pituitary dysfunction, whereas normalization of PRL might help to differentiate idiopathic short stature (ISS) from true GHD.

The aim of the study was to (i) evaluate the effect of alpha2-adrenergic receptor stimulation following clonidine administration on PRL secretion, as well as to (ii) assess differences in response to the above-mentioned stimulation in groups of children with normal GH secretion (idiopathic short stature, ISS) and in those with decreased GH secretion (GH deficiency, GHD).

## 2. Results

There were no significant differences between the groups with respect to chronological age, bone age, height standard deviation score (HSDS) or nutritional status (BMI SDS). Most of the children were prepubertal (Tanner stage 1), only seven of them were in the early stage of puberty (Tanner stage 2). As expected, children with GHD exhibited significantly lower IGF-1 SDS values and significantly lower GH concentrations at each of the consecutive time points during the stimulation test after clonidine administration (except for the 0′ point)—as determined by the GHD group definition. In contrast, no significant differences between groups were found in mean PRL concentration at individual time points during this test. Data for the groups analyzed are shown in Table 1. 

**Figure 1:**
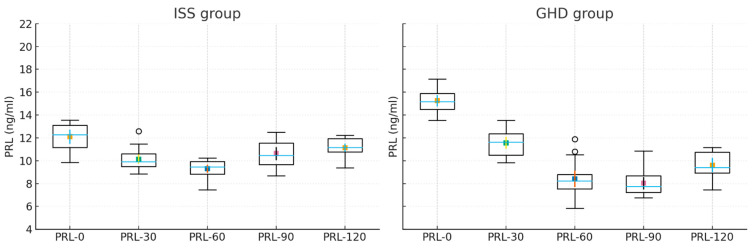
The results of PRL measurements (mean ±SD) during the individual time points of the stimulation test with clonidine in ISS and GHD.

To assess within-group changes in PRL concentrations during the clonidine test, we first applied the Wilcoxon paired test. The analysis was performed for the whole group and for individual subgroups: children with GHD and children with ISS. We found that in the analyzed group, significantly lower PRL concentrations were observed after 30′, 60′, and 90′ min compared to baseline values, and these differences were observed for the entire group and for each group separately (Table 2). While further differences depended on the diagnosis. In GHD, a subsequent significant decrease in PRL concentration was observed between time point 30′ and time points 60′ and 90′, and then PRL values did not increase significantly until the end of the test. In contrast, in ISS, the PRL reached a nadir at 30′ but then increased significantly from 60′ onward, with values at 60′, 90′, and 120′ differing significantly from the nadir and approaching baseline levels. That means that in the GHD there was no return of PRL to normal values during the test, whereas in the ISS there was a subsequent PRL recovery following the nadir at 30′. This has been presented in Table 2 and Figure 1. 

In addition, a formal repeated-measures analysis confirmed a significant effect of time (F(4, 112) = 12.34, *p* < 0.001) and a significant group × time interaction (notably at 90′, *p* = 0.005; and 120′, *p* = 0.001). This analysis demonstrates that not only mean values, but also the temporal pattern of PRL secretion during the clonidine test, differs significantly between ISS and GHD.

**Figure 2:**
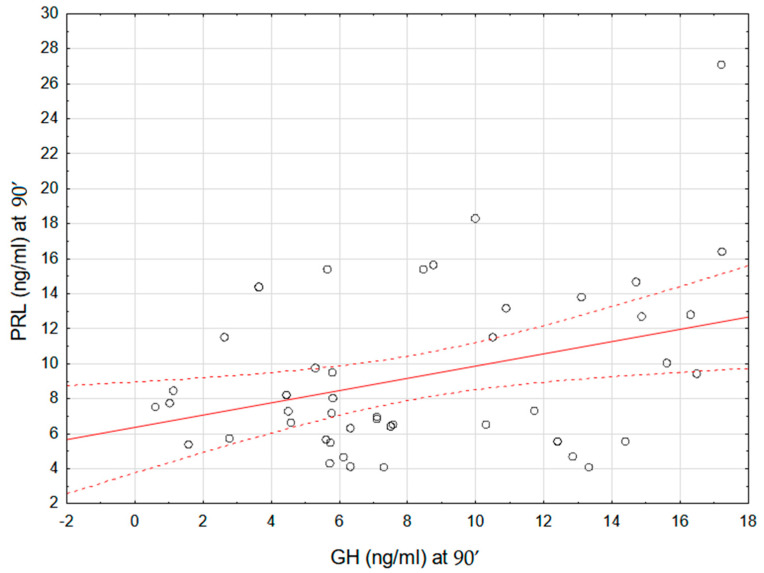
Correlation between GH and PRL concentrations at 90′ of the stimulation test after clonidine administration. Each point represents an individual subject. The solid red line shows the linear regression line, and the dashed red lines indicate the 95% confidence interval.

**Figure 3:**
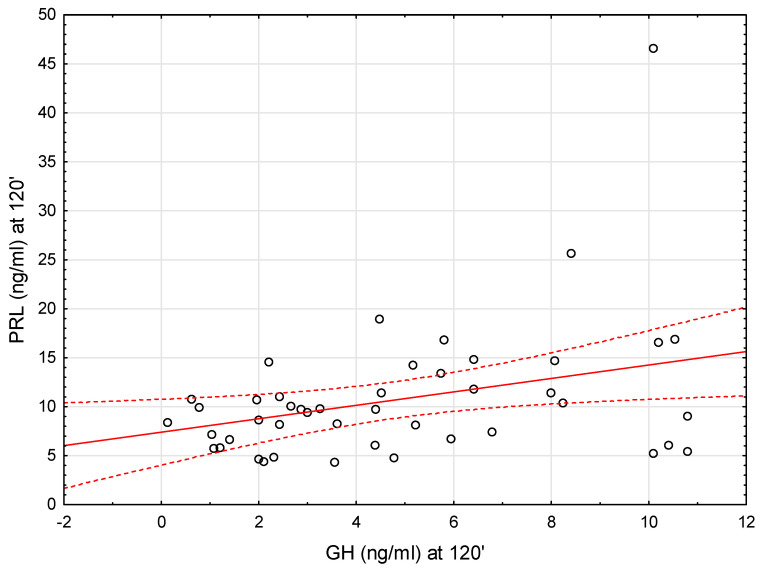
Correlation between GH and PRL concentrations at 120′ of the stimulation test after clonidine administration. Each point represents an individual subject. The solid red line shows the linear regression line, and the dashed red lines indicate the 95% confidence interval.

We therefore looked for a correlation between GH and PRL secretion during the test. We found that there was a modest positive correlation between GH and PRL concentrations at 90 min of the test (r = 0.35, *p* < 0.05) as well as PRL and GH concentrations at 120 min of the test (r = 0.35, *p* < 0.05) (see Figure 2 and Figure 3).

## 3. Discussion

PRL- and GH-secreting cells are derived from the common somatomammotropin progenitor cells in the anterior pituitary gland and can be transformed into somatotrophs or lactotrophs according to specific stimuli [12]. Furthermore, their receptors structurally belong to the family of class I cytokines rather than to classical endocrine systems such as peptides or steroids, and have extensive biological effects, as do other cytokines [6,13]. Secretion of GH depends on GHRH, somatostatin (GH-inhibiting hormone, GHIH), and ghrelin, but several other factors are undoubtedly involved in the regulation of GH secretion (e.g., thyroid hormones, hypoglycemia) [14,15]. PRL secretion is also regulated by a variety of factors, of which the hypothalamic tuberoinfundibular dopamine neurons (TIDA) located in the arcuate nucleus of the hypothalamus (ARH) have been found to be the most important [3,16]. TIDA neurons inhibit PRL synthesis and release via activation of dopamine D2 receptors in lactotrophic cells [17]. Moreover, the secretion of PRL is regulated by TRH, estrogens, and GHIH [16,18]. These two hormones (PRL and GH) have partly the same common secretory mechanisms, such as estrogens, dopamine, TRH, ghrelin, stress or hypoglycemia [3,12]. 

In our previous study, we showed a positive correlation between GH secretion in stimulation tests and nocturnal PRL secretions [19]. Thus, lower GH secretion was associated with reduced nocturnal PRL secretion. We also showed that congenital structural abnormalities of the hypothalamic-pituitary axis not only impair GH but also PRL production [20]. There were only a few studies on the concomitant measurements of PRL and GH. High secretion of both hormones has been presented in patients with Laron syndrome and in newborns [21,22]. In neonates, serum PRL and GH levels exhibited a positive correlation, which may be explained by common regulatory agents or a drift phenomenon involving somatomammotrophic cells [21]. Interestingly, Völkl et al. demonstrated that children with GHD had higher PRL levels compared to children with ISS [23]. PRL was secreted spontaneously, without requiring stimulating input [23]. 

In this study, we aimed to assess whether GH secretion during the clonidine stimulation test may, in any way, be related to PRL secretion. During the clonidine stimulation test, a significant decrease in PRL levels was observed shortly after drug administration in both the GHD and ISS groups. To our knowledge, only one study assessed both GH and PRL levels in clonidine stimulation tests in children with GHD, and their results were consistent with ours in showing that clonidine suppressed PRL. Silbergeld et al. likewise reported PRL suppression during GH stimulation tests, highlighting the reproducibility of this effect across different protocols [24]. However, unlike these earlier reports, our study also demonstrated distinct recovery dynamics between groups. In ISS, PRL levels rose back toward baseline after the nadir, whereas in GHD they remained persistently suppressed. This difference suggests that although clonidine uniformly induces an early decline in PRL, the subsequent trajectory of PRL secretion may carry additional diagnostic information

It is important to emphasize that during the GH stimulation test following clonidine administration, what is primarily being evaluated is the function of the GHRH-GH axis and the effect of noradrenergic stimulation on PRL suppression, rather than the presence and activity of somatotropic and lactotrophic cells. This distinction is crucial, as alpha2-adrenergic stimulation exerts an indirect effect on the secretion of both hormones. Actually, alpha2-adrenergic receptor agonists can be divided into three groups: phenylethylline and oxaloazepines and imidazolines—which include clonidine. Devesa et al. demonstrated that alph2-adrenergic agonism primarily acts in GH control by inhibiting hypothalamic somatostatin release, rather than stimulating GHRH secretion [25]. Whereas a study conducted by McMahon et al. suggests that alpha2-adrenergic-induced secretion of GH occurs through a dual mechanism involving both inhibition of somatostatin (GHIH) neurons in the periventricular nuclei and thereby removing tonic inhibition of somatoliberin (GHRH) neurons in the ARC as well as a direct stimulation of somatoliberin (GHRH) release from axon terminals in the median eminence [26]. The study conducted by Lien et al. indicates that the alpha2-adrenergic system also plays a role in the regulation of PRL release. They showed that clonidine administered in rats at a dose of 0.2 mg/kg reduced blood PRL levels. Furthermore, clonidine administered after pretreatment with alpha2-adrenergic receptor antagonist (yohimbine, piperoxan and Wy-26,70) reversed the release of PRL, normalizing its plasma levels [27,28]. Lien et al. also studied the effect of clonidine in lactating rats. They documented a significant reduction in PRL levels after clonidine administration [28]. However, clonidine may have different functional effects depending on the dose. It was found that clonidine at low doses stimulated presynaptic alpha2-adrenergic receptors of adrenergic neurons. Higher doses of clonidine stimulated postsynaptic α-adrenergic receptors. Low doses (0.05 mg and 0.2 mg) of clonidine inhibited PRL, which was also confirmed in our study, while a higher dose (1 mg/kg) did not change the baseline PRL concentration. In contrast, a very high dose (5 mg/kg) led to the release of PRL into the plasma [28].

Our results demonstrate distinct patterns of PRL response to clonidine in children with ISS and GHD. While both groups showed an initial decline in PRL after 30 min, in ISS this was followed by a significant recovery toward baseline, whereas in GHD, PRL levels remained suppressed without subsequent normalization. The repeated-measures analysis confirmed a significant group-by-time interaction, indicating that the temporal pattern of PRL secretion differs between the two groups. This observation may be attributed to a few potential factors: (i) reduced functional activity of GH- and PRL-secreting cells in children with GHD, (ii) a prolonged phase of alpha2-adrenergic stimulation in children diagnosed with GHD, (iii) involvement of growth hormone receptor (GHR) signaling in PRL release. Recently, Wasinski et al. demonstrated that GH action in dopaminergic neurons is required for stress-induced PRL release in male mice [29]. Their study suggests that GHR signaling in dopaminergic neurons may be involved in the regulation of PRL secretion.

Taken together, while PRL monitoring during clonidine testing cannot by itself discriminate ISS from GHD, the distinct recovery patterns may serve as a supportive marker in cases with borderline GH results and should be considered hypothesis-generating, pending validation in larger cohorts. This study has several important limitations that should be considered when interpreting the results. First, the relatively small sample size represents a major limitation and may restrict the generalizability of the findings. Although the statistical power was sufficient to detect the main effects, the limited number of participants increased the risk of both type I and type II errors, potentially obscuring more subtle associations. Second, circadian variation and pubertal status, both well-established determinants of PRL secretion, could have acted as residual confounders despite the use of standardized testing conditions. All participants were prepubertal or in early puberty, which reduces the potential for bias related to pubertal stage. The small sample size precluded stratified analyses by pubertal status and sex, which would have allowed a more detailed assessment of these effects. Finally, the findings have not yet been replicated in an independent cohort. Validation in larger and more diverse populations is necessary to confirm the robustness and external validity of these observations.

## 4. Materials and Methods

The study included consecutive children who were admitted to the Department of Endocrinology and Metabolic Diseases of the Polish Mother’s Memorial Hospital—Research Institute in Lodz over aa 6-month period for the diagnosis of their short stature.

The height (measured using a stadiometer) and body mass were assessed in all children, and that was followed by calculation of the height standard deviation score (HSDS) and body mass index SDS (BMI SDS) according to Polish centile charts [30]. Only children with HSDS below −2.0 from the mean value for the child’s age and sex were qualified into the study group. The stage of puberty was assessed in each child by Tanner’s scale. From study group we excluded the children with known genetic reasons of the short stature (i.e., Turner syndrome, Prader–Willi syndrome) assessed based on a karyotype as well as the children with untreated hypothyroidism, chronic diseases or undiagnosed gastrointestinal tract complaints (evaluated based on a negative history of chronic diseases, as well as normal tests results of tissue transglutaminase antibodies class IgA). 

Finally, we analyzed the group of 49 children (mean age ± SD: 9.55 ± 3.48 years), who met the above-mentioned criteria. In accordance with national recommendations, two stimulation tests (clonidine and glucagon) were performed in all participants to enhance diagnostic reliability [9]. All stimulation tests were conducted in the morning under fasting conditions to minimize circadian and nutritional effects, and the standardized hospital setting ensured uniform procedures, limiting variability from stress or anxiety. The first stimulation test was performed after oral administration of clonidine (a stimulator of α_2_-adrenergic receptors responsible for GHRH production and secretion) at a dose of 0.15 mg/m^2^ of body surface area, with GH concentrations measured at baseline (0 min) and at the 30th, 60th, 90th, and 120th minute of the test. The second stimulation test was performed after intramuscular administration of glucagon at a dose of 30 µg/kg of body weight (not exceeding 1 mg), with GH concentrations measured at baseline (0 min) and at the 90th, 120th, 150th, and 180th minute of the test. 

Although the diagnostic threshold for GHD is a matter of debate internationally, in Poland, the diagnostic cut-off for GHD is a peak GH concentration < 10 ng/mL [31]. 

Therefore, based on the results of GH_max_ values in these tests, we diagnosed:

- ISS group—children with normal GH secretion (GHmax values ≥ 10 ng/mL in at least one test); *n* = 29 children
- GHD group—children with decreased GH secretion (GHmax values < 10 ng/mL in both tests); *n* = 20 children.

In the GH secretion stimulation test after oral clonidine administration, in addition to GH concentrations, serum PRL concentrations were also assessed at the same individual time points. In each child the concentration of IGF-1, IGFBP-3, TSH, FT4 and FT3 was also assessed in the fasting state on the first day of hospitalization, just before the first stimulating test. Next, IGF-1 concentrations were calculated as IGF-1 SDS, according to the reference data [31]. For the calculation of IGF-1/IGFBP-3 molar ratio, the following molecular masses were used: 7.5 kDa for IGF-1 and 42.0 kDa for IGFBP-3. For IGF-1/IGFBP-3 molar ratio. Bone age was assessed in all children included in the study using radiographs of the carpal and non-dominant hand bones according to the G&P method. In each child with GHD, an MRI of the pituitary gland was also performed. Apart from 2 children who were diagnosed with pituitary stalk interruption syndrome (PSIS), no pathology in this organ was found in the others. These children were excluded from further studies.

PRL concentrations were measured by the electrochemiluminescence method on the Roche Elecsys^®^ Systems (Roche Diagnostics GmbH, Mannheim, Germany). The assay had asensitivity 0.47 ng/mL, and a measurement range of up to 470 ng/mL. The inter-assay coefficient of variation (CV) was 1.8–3.4%. GH levels were measured using the immunometric method with Immulite assay kits (Diagnostic Products Corporation, Los Angeles, CA, USA). The assay was calibrated to the WHO IRP 98/574 standard set. The sensitivity was 0.01 ng/mL, range: up to 40 ng/mL, the conversion index: ng/mL × 2.6 = mIU/L, the intra-assay CV: 5.3–6.5% and inter-assay CV: 5.5–6.2%. Both IGF-1 and IGFBP-3 concentrations were measured using Immulite assay kits (Diagnostic Products Corporation, Los Angeles, CA, USA). For IGF-1, the assay was calibrated against the WHO NIBSC 1st IRR 87/518 standard, with the analytical sensitivity of 20 ng/mL, calibration range up to 1600 ng/mL, the intra-assay CV: 3.1–4.3% and inter-assay CV: 5.8–8.4%. The assay for IGFBP-3 assessment was calibrated to the WHO NIBSC Reagent 93/560 standard, with analytical sensitivity 0.02 μg/mL, the calibration range up to 426 μg/mL, the intra-assay CV: 3.5–5.6% and the total CV: 7.5–9.9%.

The data were analyzed using Statistica version 13.3 software (StatSoft, Inc., Tulsa, OK, USA). The continuous variables were expressed as mean ± standard deviation for normally distributed variables. Shapiro–Wilk’s test was used to assess the distribution of the variables. Between-group comparisons were carried out with Student’s *t*-test or Mann–Whitney U test, as appropriate. Within-group changes in PRL concentrations across time points during the clonidine test were assessed using the Wilcoxon signed-rank test. To evaluate whether PRL trajectories differed between ISS and GHD, a repeated-measures ANOVA (group × time interaction) was performed. Correlations between GH and PRL concentrations at individual time points were examined using Pearson’s correlation coefficient. A two-sided *p* < 0.05 was considered statistically significant.

## 5. Conclusions

Our findings reveal that clonidine-induced alpha2-adrenergic receptor stimulation leads to transient suppression of PRL levels in both ISS and GHD children, whereas only ISS patients demonstrate a recovery of PRL levels. The difference in patterns observed between GHD and ISS suggests a possible role of GH signaling in PRL regulation. 

These findings provide new insights into the neuroendocrine mechanisms linking GH and PRL secretion and may contribute to a better understanding of hormonal responses to alpha2-adrenergic stimulation. Further research in larger, well-characterized cohorts is needed to clarify the functional implications of these interactions and their potential clinical relevance in endocrine disorders. 

## Figures and Tables

**Table 1:** Characteristics of the study groups and GH and Prl concentrations at individual time points during the stimulation test after clonidine administration.

Parameter	GHD, n = 20	ISS, n = 29	*p*-Value
Chronological age (years)	10.78 ± 2.79	9.39 ± 3.60	0.173
HSDS	−2.26 ± 0.95	−2.46 ± 1.12	0.579
BMI (kg/m^2^)	16.82 ± 2.70	16.45 ± 3.62	0.732
TSH (mIU/mL)	2.51 ± 1.51	2.51 ± 1.01	0.999
FT4 (ng/mL)	1.37 ± 0.18	1.21 ± 0.20	**0.009** *
FT3 (ng/mL)	4.09 ± 0.65	4.09 ± 0.50	0.980
IGF-1 (pg/mL)	104.13 ± 49.18	158.49 ± 103.67	0.050
IGF-1 SDS	−2.42 ± 1.27	−1.01 ± 0.70	**0.001** *
IGFBP-3 (ng/mL)	3.87 ± 1.38	4.52 ± 1.50	0.161
IGF-1/IGFBP-3 molar ratio	0.15 ± 0.04	0.18 ± 0.08	0.066
Bone age (years)	8.89 ± 3.18	8.16 ± 4.02	0.531
GH-0′ (ng/mL)	0.94 ± 1.52	1.50 ± 2.25	0.358
GH-30′ (ng/mL)	1.35 ± 1.86	3.03 ± 3.17	0.050
GH-60′ (ng/mL)	4.77 ± 3.29	12.22 ± 6.02	**0.001** *
GH-90′ (ng/mL)	4.58 ± 2.06	10.90 ± 4.32	**0.001** *
GH-120′ (ng/mL)	2.67 ± 1.70	6.30 ± 3.21	**0.001** *
PRL-0′ (ng/mL)	15.81 ± 10.73	13.34 ± 5.29	0.310
PRL-30′ (ng/mL)	10.63 ± 6.26	9.85 ± 4.16	0.618
PRL-60′ (ng/mL)	7.90 ± 3.20	8.88 ± 3.62	0.359
PRL-90′ (ng/mL)	7.68 ± 3.25	10.39 ± 5.31	0.059
PRL-120’ (ng/mL)	8.44 ± 3.45	12.34 ± 8.37	0.069

* *p* < 0.05 is bolded. ISS—idiopathic short stature; GHD—growth hormone deficiency; HSDS—high standard deviation score; BMI—body mass index; TSH—thyroid-stimulating hormone; FT4—free thyroxine; FT3—free triiodothyronine; IGF-1—insulin-like growth factor 1; IGFBP-3—insulin-like growth factor binding protein 3; GH—growth hormone, PRL—prolactin.

**Table 2:** The statistical differences between the individual measurements of PRL concentrations during the stimulation test with clonidine in both subgroups and in the total group of analyzed children.

	GHD, *p*-Value	ISS, *p*-Value	Total Group, *p*-Value
PRL-0 & PRL-30	**0.001** *	**0.001** *	**0.001** *
PRL-0 & PRL-60	**0.001** *	**0.001** *	**0.000** *
PRL-0 & PRL-90	**0.005** *	**0.019** *	**0.001** *
PRL-0 & PRL-120	**0.022** *	0.151	**0.011** *
PRL-30 & PRL-60	**0.006** *	0.058	**0.001** *
PRL-30 & PRL-90	**0.043** *	0.904	0.221
PRL-30 & PRL-120	0.396	0.113	0.463
PRL-60 & PRL-90	0.896	**0.012** *	**0.041** *
PRL-60 & PRL-120	0.199	**0.001** *	**0.001** *
PRL-90 & PRL-120	0.084	**0.001** *	**0.001** *

* *p* < 0.05 is bolded. ISS—idiopathic short stature; GHD—growth hormone deficiency; PRL—prolactin.

## Data Availability

The data presented in this study are available on request from the corresponding author.
